# Meta-analysis of the functional neuroimaging literature with probabilistic logic programming

**DOI:** 10.1038/s41598-022-21801-4

**Published:** 2022-11-12

**Authors:** Majd Abdallah, Valentin Iovene, Gaston Zanitti, Demian Wassermann

**Affiliations:** Inria, CEA, Neurospin, MIND Team, Université Paris Saclay, 91120 Palaiseau, France

**Keywords:** Cognitive neuroscience, Data integration, Data mining, Data processing, Programming language, Statistical methods

## Abstract

Inferring reliable brain-behavior associations requires synthesizing evidence from thousands of functional neuroimaging studies through meta-analysis. However, existing meta-analysis tools are limited to investigating simple neuroscience concepts and expressing a restricted range of questions. Here, we expand the scope of neuroimaging meta-analysis by designing NeuroLang: a domain-specific language to express and test hypotheses using probabilistic first-order logic programming. By leveraging formalisms found at the crossroads of artificial intelligence and knowledge representation, NeuroLang provides the expressivity to address a larger repertoire of hypotheses in a meta-analysis, while seamlessly modeling the uncertainty inherent to neuroimaging data. We demonstrate the language’s capabilities in conducting comprehensive neuroimaging meta-analysis through use-case examples that address questions of structure-function associations. Specifically, we infer the specific functional roles of three canonical brain networks, support the role of the visual word-form area in visuospatial attention, and investigate the heterogeneous organization of the frontoparietal control network.

## Introduction

Meta-analysis confronts hypotheses of brain-behavior mappings with synthesized evidence from thousands of functional neuroimaging studies^1^. However, commonly used standard tools for meta-analysis, such as BrainMap^2^ and Neurosynth^3^ are limited in their *formal expressivity*. For example, it is challenging to express complex questions like ‘which topics are likely to be present in a study given activation in one set of regions and *there exists no* activation in another set of regions’. Answering this question requires that we explicitly run a meta-analysis on every single term of interest, while integrating heterogeneous and uncertain data, such as spatial masks and parcellations, to define the regions^4–7^. Importantly, however, applying the nontrivial criteria to select articles for inclusion and exclusion necessitates coding in a general-purpose programming language, which can be error-prone and hard to maintain. In this work, we harness advancements in symbolic artificial intelligence, specifically in *probabilistic logic programming*, to design *NeuroLang*: a domain-specific language (DSL) to formulate rich and expressive neuroscience hypotheses using formal logic-based criteria while seamlessly combining heterogeneous data and reasoning about uncertainty. Our bet is that NeuroLang’s clear syntax and probabilistic logic semantics will enable accessible, rigorous, and highly reproducible large-scale meta-analysis.

Large-scale meta-analysis has become popular in cognitive neuroscience research as a result of the growth in non-invasive neuroimaging experiments, which provoked an upsurge in the number of yearly publications on structure-function associations^8^. As a powerful approach to synthesizing large quantities of results, meta-analysis has been performed to distinguish spurious from replicable findings, derive coactivation patterns^9^, and define a priori regions of interest^10^. Moreover, elaborate coactivation-based connectivity, meta-analytic functional parcellations, and sophisticated network models are now being inferred from datasets covering tens of thousands of subjects. Recently, even more, elaborate meta-analyses have been performed to derive new findings. For instance, Yeo et al.^6^ have used meta-analysis to study the human association cortex, identifying frontal and parietal regions that are either specialized or flexible. Furthermore, meta-analysis has been used to support findings on the fractionation of networks into subsystems underlying disparate processing domains^7,11^. Thus, large-scale meta-analysis is essential for the continued detention of latent properties of brain systems, far beyond what can be inferred from individual studies.

Over three decades ago, spatial normalization—the use of peak activation coordinates standardized in stereotaxic space—was introduced to human neuroimaging research^12^. This norm has been embraced, enlarged, and popularized by an outstanding series of methodological breakthroughs in the field. As a result, a large and methodologically cohesive functional neuroimaging literature has emerged over the past years. Capitalizing on this wealth of results, large-scale online databases have been created to compile peak activation coordinates and their related meta-data, spurring the development of coordinate-based meta-analytic approaches to exploit this rapidly growing corpus. This makes coordinate-based meta-analysis (CBMA) the most popular type of neuroimaging meta-analysis so far. The earliest approach to compiling peak coordinates, used by BrainMap^2^, is to manually transcribe them from study tables. The peaks are then linked to the content of the studies by taxonomy experts. A more recent approach, used by Neurosynth^3^, is to automate this laborious task via special software that automatically extracts peak coordinates from study tables. Neurosynth also automates the annotation of studies using natural language processing techniques that incorporate term-frequency features, i.e. TF-IDF, estimated from the texts of studies. Albeit noisier, these fully automated approaches to meta-analysis scale better to the rapidly expanding neuroimaging literature.

Tools like BrainMap^2^ and Neurosynth^3^ have indeed simplified meta-analysis, becoming cornerstones of contemporary neuroscience research. However, only a narrow range of questions can be *natively* posed using these tools due to their limited *expressivity*. Specifically, these tools are based on propositional logic, which makes formulating queries beyond straightforward propositions, while applying nontrivial selection criteria of studies, a verbose and arduous task. More recently, NeuroQuery, a regularised predictive model trained on CBMA data, has been introduced to map *any arbitrary text fragment* (i.e. a set of keywords of interest, dubbed ‘query’) to a brain activation map. This approach shows that modeling semantic relations across studies can produce meaningful statistical maps for terms that are rarely mentioned in the literature. Nonetheless, NeuroQuery cannot express questions that infer a pattern of activation from studies associated with “working memory”, for instance, *but not associated* with other related functions. Such an analysis could be interesting to infer unique neural substrates among closely-related mental functions. Yet, NeuroQuery does not employ any *logic-based semantics*. That is, the words ‘but’ and ‘not’ are not in NeuroQuery’s pre-selected vocabulary, and have no influence on the resulting statistical maps. Thus, there seems to be a space for a logic-based framework that, by giving access to more elaborate meta-analytic queries, could help bridge the gap between statistical modeling and cognitive neuroscience. An all-encompassing domain-specific language (DSL) for neuroscience research could encode any type of data, express complex queries using logic semantics, and reason about elements of uncertainty, all in a single unifying framework.

Parallel to the growth of functional neuroimaging over the past few decades, the artificial intelligence (AI) sub-fields of *probabilistic logic programming* and *probabilistic databases* have experienced a rapid evolution. This has led to the development of methods that efficiently represent knowledge with both *logical* and *probabilistic* semantics in a the way that makes statistical model assumptions declarative, clear, and less biased. Theoretical formalisms ground probabilistic logic languages with well-defined semantics and a mathematical understanding of the complexity of various classes of queries and different types of data^13^. Efforts to address the scalability of probabilistic logic systems led to recent algorithms and data structures that can remarkably speed up analyses, even on very large and diverse databases^14^.

Can probabilistic logic programming formalize and broaden the range of questions that can be expressed in a meta-analysis? Can it simplify formulating complex hypotheses while combining heterogeneous and uncertain data? In this work, we present NeuroLang, a domain-specific language for conducting comprehensive neuroimaging meta-analyses using formal, succinct, and self-contained programs. At its core, NeuroLang uses predicate logic, as opposed to propositional logic, to ease the formulation of queries in a way that is closer to human discourse^15^, and that can be run against structured neuroscientific data. Logic rules and queries are augmented with probabilistic semantics to account for the uncertainty that emerges from missing information, analytical variability across studies, and measurement imperfections. In this article, we present concrete use-case applications of NeuroLang that address popular questions from the literature.

## Results

The use-case examples of NeuroLang shed light on the utility of probabilistic logic semantics in representing neuroscience hypotheses that cannot be readily expressed with standard meta-analysis tools. In the first example, we use between-network segregation queries to infer the unique functional roles of three canonical functional networks: the dorsal attention network (DAN), default mode network (DMN), and frontoparietal cognitive control network (FPCN). In the second example, we explore potential associations between topics and activity within the visual word-form area (VWFA), when it either coactivates with regions of the dorsal attention network or those of the language network. In the third and fourth examples, we study the functional heterogeneity of the FPCN, uncovering differential activation profiles for a number of mental functions and varying connectivity patterns with other brain networks.

### Representing neuroscientific knowledge under uncertainty

Before exploring use-case examples of NeuroLang, we describe how heterogeneous neuroimaging data are represented by using fact and rule *tables*. A table is a set of tuples or rows, each representing a data instance and having a set of *k* elements representing columns. *Probabilities* can be ascribed to the rows of a table to quantify the level of uncertainty in the data presented by each, in which case the table is said to be *probabilistic*.

Studies in a CBMA database report a set of *peak* activations that we store in a table named PeakReported. This table contains one row (*x*, *y*, *z*, *s*) for each peak that a study *s* has reported at location (*x*, *y*, *z*) in the Montreal Neurological Institute (MNI) standard space. Moreover, the uncertainty around the spatial location of peaks can be encoded in a rule table by assuming each peak’s 10 mm neighboring voxels to be equivalently reported, similar to the multilevel kernel density analysis (MKDA)^16^. This rule table is called VoxelReported, and it includes a row (*x*, *y*, *z*, *s*) for each voxel at location (*x*, *y*, *z*) within a radius ($r<10$ mm) of a peak reported by study *s*. The choice of using a 10 mm radius is consistent with the smoothing radii commonly used in the functional neuroimaging literature^17^. More details on how other spatial smoothing priors can be encoded in NeuroLang, such as the probabilistic prior used by the activation likelihood estimation (ALE)^18^ algorithm, are provided in the Supplementary Materials.

**Figure 1:**
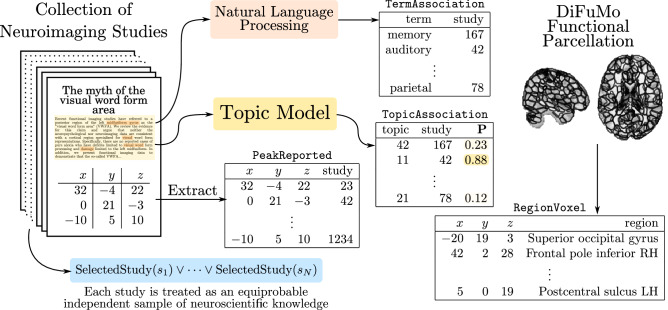
Representation of meta-analytic and functional parcellation knowledge using database tables in NeuroLang.

Further, each study within a meta-analytic corpus is associated with cognitive processes or concepts addressed by its experiments. Fully automated meta-analytic tools like Neurosynth calculate statistical term-frequency features on study texts or abstracts, and threshold them to establish these links in a data-driven manner^3^. We store these associations within a TermAssociation table, containing one row (*t*, *s*) for each term *t* associated with study *s*. Moreover, we incorporate data-driven topic models, learned and openly shared by Neurosynth^19^, within a TopicAssociation probabilistic table, containing one row $(t, s,\mathbf{P} )$ for each uncertain association between a topic *t* and a study *s*. In probabilistic logic, we write $\texttt {TopicAssociation}(t, s) {:}{:} \mathbf{P}$ to state ‘study *s* has a probability **P** of being associated with topic *t*’^20^. This data representation process is illustrated in Fig. 1.

Similarly to Neurosynth, we assume each study within the meta-analytic database to be an *independent equiprobable sample* of neuroscientific knowledge^3,18^. This assumption is encoded by a SelectedStudy probabilistic table, depicted in the bottom left part of Fig. 1, which gives studies an equal weight 1/*N* in any meta-analysis, where *N* is the total number of studies within the meta-analytic database. This makes it possible to estimate statistics on CBMA databases in the absence of statistical power indicators (e.g., sample size).

It is common for meta-analyses to integrate anatomical or functional brain parcellations^11^ to enhance interpretability and reduce computational burdens. In the examples, we use the DiFuMo-256 atlas^21^, which is part of a multiscale “soft” parcellation estimated from thousands of subjects across 27 studies that include both task and resting-state fMRI experiments. This data-driven functional atlas is argued to achieve comparable statistical performance as voxel-level analyses while simultaneously reducing computational cost and enhancing interpretability. We represent the 256 functional regions from DiFuMo in a RegionVoxel table, containing a row (*r*, *x*, *y*, *z*) for each brain voxel at MNI location (*x*, *y*, *z*) belonging to a DiFuMo-256 region *r*. An excerpt of the RegionVoxel table is depicted on the right part of Fig. 1. We also incorporate a table NetworkRegion that contains a row (*n*, *r*) for each region *r* that significantly overlaps with some network *n* from the 7 or 17-network parcellations^22^. The network membership of regions is provided as part of the DiFuMo meta-data file. The experiments that follow will use this unified framework of knowledge representation to express probabilistic logic programs that drive meta-analytical findings.

### Between-network segregation: reverse inference of brain network function

In this example, we perform a *segregation-based meta-analysis* to infer the likelihood of a topic to be present in a study given activation in a brain network, with an additional constraint that there *exists no activation in other networks*. The goal of this example is to show that a segregation query can identify which network’s activation pattern is preferentially more predictive of the presence of topic terms related to certain mental functions.

We use the Neurosynth CBMA database^3^, consisting of 14,371 studies, and its associated v5-topics-100 topic model^19^. The networks included in this example are the DMN, FPCN and DAN defined using the coarse 7-Network atlas^22^. These networks exhibit coupling dynamics in support of an array of internally and externally-directed mental functions^23^. However, each one of them is believed to subserve a unique set of cognitive processes^7,23,24^. The FPCN contributes to a wide variety of tasks by engaging top-down control processes, the DAN is concerned with orienting attention towards salient cues, and the DMN is involved in abstract self-referential, social and affective functions. Using a segregation query, we can quantitatively identify the specific functional roles of these networks from the literature.

First, we have to represent useful heterogeneous data in NeuroLang. For instance, we assume a DiFuMo-256 component *r* to be reported by a study *s* whenever a peak activation is reported by the study within that region. In NeuroLang, this is expressed by the following logic rule
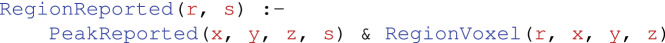
which translates, in plain English, to ‘region *r* is reported by study *s* if *s* reports a peak at location (*x*, *y*, *z*) that falls within region *r*’. All letters in this code represent variables. Furthermore, we model the reporting of networks by studies in a *probabilistic* table. The probabilities are based on the total volume of the reported regions that belong to a network. This table accounts for the uncertainty in the location of reported peak activation coordinates as well as the number of potentially reported regions. More precisely, we consider that each study has a probability of reporting a network, proportional to the number of reported regions belonging to the network.

This is implemented by the following rules in NeuroLang
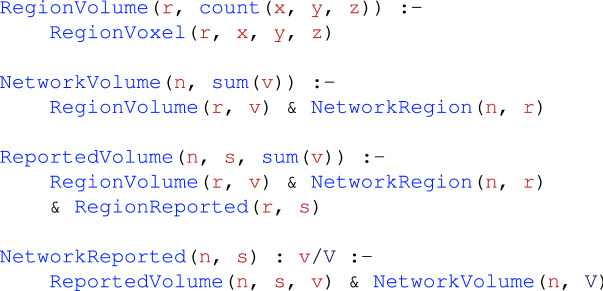


In plain English, a network *n* is considered to be reported by study *s* with probability *v*/*V*, where *v* is the total volume of regions within network *n* that are reported active by study *s*, and *V* is the total volume of all regions in the network. This program makes use of NeuroLang’s ability to express probabilistic rules -i.e. NetworkReported-, aggregations via the built-in count and sum functions, and probabilistic inference capabilities.

Next, we define a rule that infers the probability that studies are associated with a topic *given* activation in only one of the three networks. That is, we query the probability that a topic *t* reported by study *s* is associated with activation in some network *n* reported by *s* and *there exists* no other network reported by study *s*. In NeuroLang, this corresponds to the following rule that infers the probability of preferential association between a topic and a network across the whole dataset:
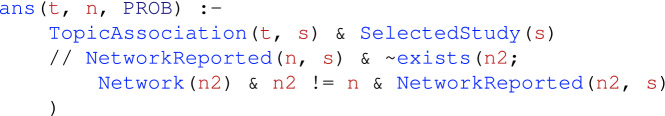
where the // operator is read as *given*, representing *probabilistic conditioning*. This rule contains a negated existential expression, $\sim$exists($\cdots$), that prevents two or more networks from being reported by a study at the same time. NeuroLang only allows stratified negation^25^. For a detailed description of Neurolang’s semantics, please refer to Zanitti et al.^26^.

**Figure 2:**
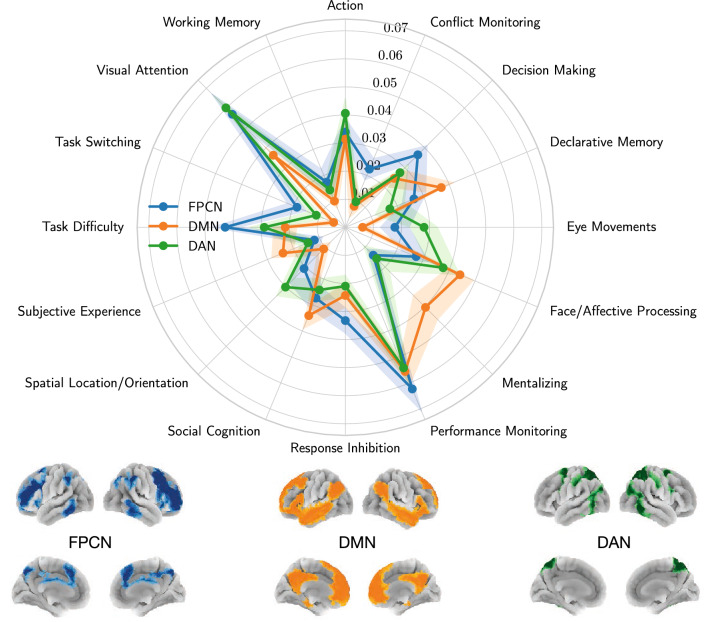
Functional profiles obtained with network-based segregation queries that identify the most probable topic associations in studies reporting activations within one network but not reporting activations within any of the other networks. A 95% confidence interval is depicted, across 1000 random 50% sub-samples of the Neurosynth database^3^. The three networks are depicted in the bottom panel of the figure.

We report the resulting functional profiles in Fig. 2. We observe that topics related to sensory processing of direct environmental demands such as eye movements, visual attention, and spatial orientation are more likely to appear in studies reporting activations in the DAN only. Also, we observe that topics related to cognitive control such as task switching, task demands, response inhibition, and performance monitoring are more likely to be mentioned in studies reporting activations in the FPCN. Finally, topics related to higher-order abstract cognitive and memory-related processes are mostly associated with studies reporting DMN activations only. Each probability value represents a ratio of the number of studies in which a topic is reported alongside an activation in only one network to the total number of studies that report activation only in that network.

### Meta-analysing the role of the visual word-form area in attention circuitry

The visual word-form area (VWFA) has attracted controversy over the years with recent findings suggesting it takes part in the attention circuitry not only in the language network^27^. Can this relationship be inferred solely from a meta-analysis of past studies that have reported activations in the left ventral occipitotemporal cortex without necessarily identifying it as the VWFA?

To answer this question, we write queries that infer the most probable topic associations among studies that report activations close to the VWFA region, while simultaneously reporting activations within regions of the attention network, but *not* reporting activations within regions of the language network.

To define regions corresponding to the VWFA, the dorsal attention and language networks, we use locations defined by Chen et al.^27^ and store them in a RegionSeedVoxel table. This table contains a row (*x*, *y*, *z*, *r*) for each region *r*’s seed location (*x*, *y*, *z*). A database table NetworkRegion contains rows (*n*, *r*) for each region *r* belonging to network *n*. A brain region is considered to be reported by a study if it reports a peak activation within $10\,\text {mm}$ of the region seed location. The choice of a $10\,\text {mm}$ radius was used to facilitate comparisons with the range of smoothing kernels that are typically used within meta-analyses. This is expressed in NeuroLang as:
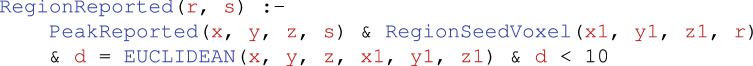
where EUCLIDEAN is a built-in function that calculates the Euclidean distance between two locations in MNI space, and thus its value is assigned to a variable; The decision to use a function application and equality to express the EUCLIDEAN builtin function is purely a syntactic choice in order to make built-in-generated values readable. Furthermore, in this example, a network is considered to be reported by a study if it reports one of the network’s regions. In NeuroLang, this rule is:
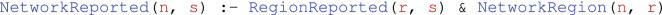


Finally, to test our hypothesis, we use the following *probability encoding rule*
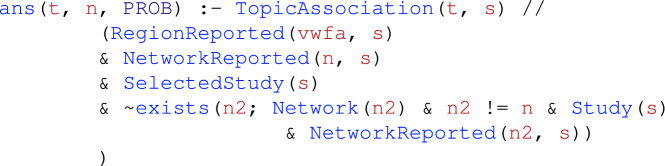
which calculates the probability of finding an association with topic *t* among studies that report the activation of both the VWFA and network *n*, but do not report the activation of any other network $n_2$, where $n_2 \ne n$. Because only two networks, language and attention, are present in the Network table, this rule simultaneously calculates the probabilities for each pair of networks, including one while segregating the other.

**Table 1:** Topics associated with studies reporting the VWFA and the frontoparietal attention network, but not reporting the ‘language’ network.

Topic	Likelihood ratio test
32_object_objects_visual	$\chi ^2(1, N = 455) = 15.96, p_{{\text {FDR}}} = 0.0065$
21_reading_words_word	$\chi ^2(1, N = 455) = 14.4, p_{{\text {FDR}}} = 0.0074$

We only depict associations surviving a likelihood ratio test and a false discovery rate (FDR) correction for multiple comparison ($p_\text {FDR} < 0.05$). More details on the likelihood ratio test are provided in the Supplementary Materials. Topics are those from Neurosynth’s v5-topics-100 topic model. Out of Neurosynth’s ${1.4371}\times 10^{4}$ studies, 455 report activations within the frontoparietal attention network without reporting activations within the ‘language’ network.

Results are shown in Table 1. Topic 32 was found to be significantly associated with studies that report activations within the VWFA and the attention network but that do not report activations within the ‘language’ network. This topic loads on terms related to *object recognition*—a task for which attention circuitry is essential^28^. This result suggests that the VWFA may play a role in attention, as studies that report its activations are significantly associated with object recognition, and supports the running hypothesis that the VWFA plays a role in processing multiple categories of visual stimuli^27^. We also observe a significant association with topic 21, which loads on terms related to the task of *reading words*—the putative role of the VWFA.

The *opposite* segregation query selects studies reporting the VWFA and the ‘language’ network but not reporting the attention network ($N = 318$). This analysis did not yield any significant topic association after correction for multiple comparisons. However, a similar topic association analysis, but without segregating studies that report activation in the attention network, does yield a significant association with topic 21, linked to the ‘reading words’ ($\chi ^2(1, N =852) = 56.86, p_\text {FDR} = 0.000081$). This result might have more than one explanation, but a plausible explanation could be the relative decrease in statistical power (i.e. smaller number of studies) in the segregation query compared to the non-segregation query.

### Inferring differential activation patterns within the FPCN using topic segregation queries

In this example, we perform forward inference using *topic-based segregation queries* to derive activation patterns within the frontoparietal cognitive control network (FPCN). As a major part of the *multiple demand* system^29^, the FPCN is associated with a large set of tasks, themselves belonging to disparate and overlapping cognitive processes such as working memory, memory retrieval, task switching, and semantic processing, to name a few. Moreover, there is evidence for a heterogeneous internal organization in the FPCN, whereby a different combination of regions may be involved in a different domain of control processing^30^. Thus, the goal of this example is to infer activation patterns within the FPCN predicted by the presence of topic terms related to one process and the simultaneous absence of topic terms related to other processes. In this sense, segregation queries can enhance the relative specificity of meta-analytic forward inferences by minimizing the amount of overlap amongst related topics.

**Figure 3:**
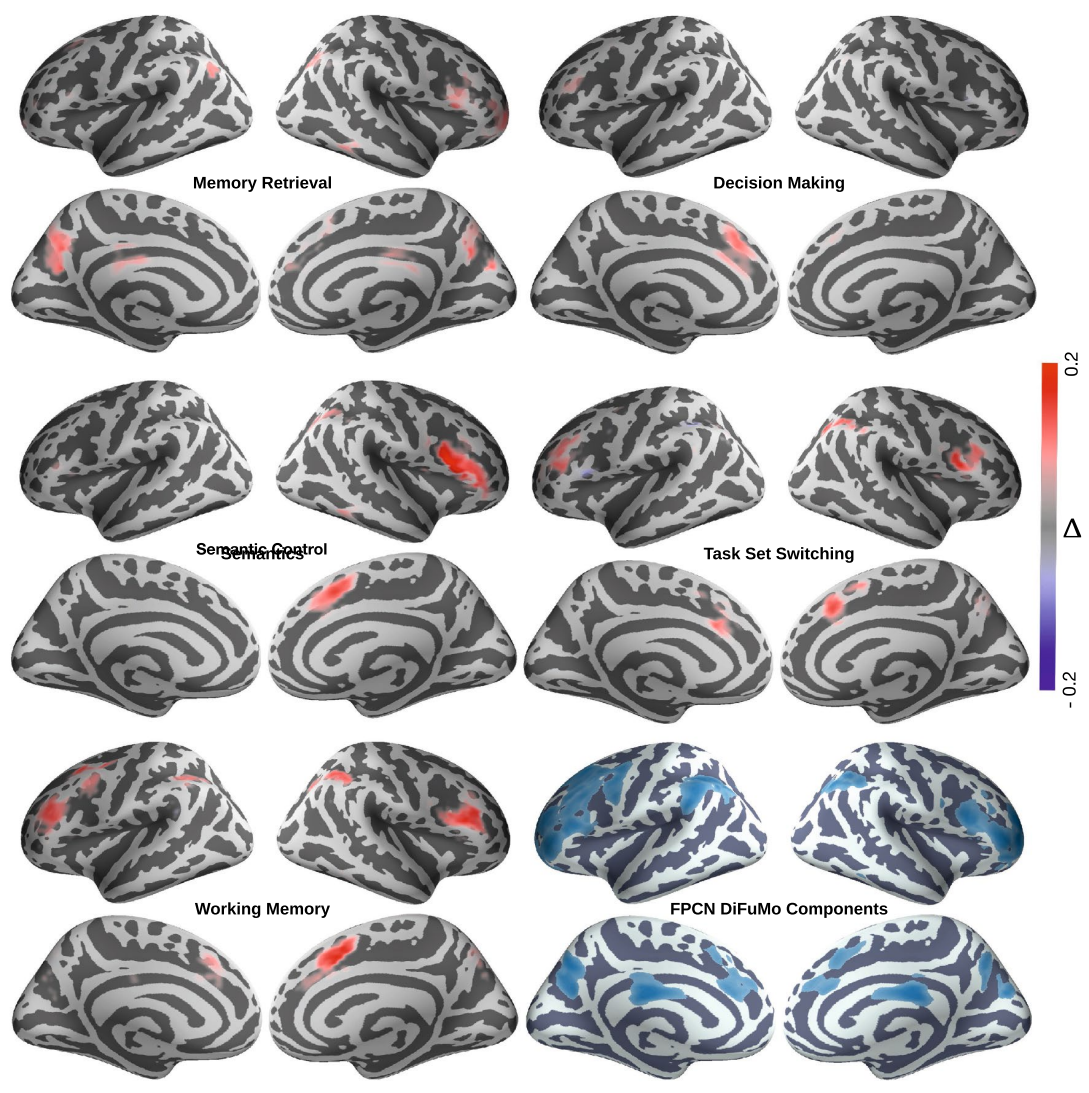
Cortical maps showing the difference in posterior probabilities of FPCN regions to be active given topic segregation and when given no topic segregation queries. We mask out brain voxels that are not part of the FPCN. The difference between posterior probabilities is defined as $\Delta = \mathbf{P} [ \text {VoxelReported}(x, y, z) | \text {SingleTopicAssociation}(t) ] - \mathbf{P} [ \text {VoxelReported}(x, y, z) | \text {TopicAssociation}(t)]$.

From the set of 200 Neurosynth topics (version-5), we select five exemplar topics representing a subset of the cognitive processes often attributed to the FPCN, along with the loading values of studies on each topic. These topics are *working memory*, *decision making*, *task set switching*, *semantic control*, and *memory retrieval*^23,29,31,32^. Then, we express the following NeuroLang program which performs topic segregation queries, yielding an activation map for each topic separately. This program is written as follows:
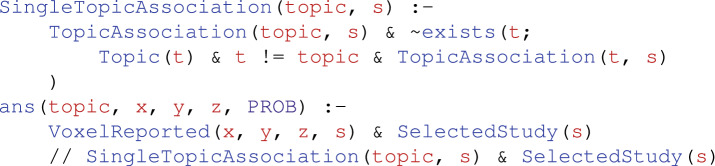


We report the resulting topic-based activations within the FPCN in Fig. 3. The results of this segregation query show that the FPCN exhibits a varied activation profile across topics, corroborating previous findings of flexible adaptation of activity within this network as task demands change. Specifically, working memory and task set switching tends to activate, to some extent, spatially interleaved, frontal, and parietal regions of the FPCN network. Semantic processing, on the other hand, dominantly activates a left-lateralized ventral frontal regions. Finally, decision making and memory retrieval are associated with activation in the cingulo-medial portion of the FPCN, the pre-supplementary motor/dorsal anterior cingulate cortex (decision making), and a precuneus/posterior cingulate cortex network (memory retrieval).

### Inferring varying meta-analytic connectivity profiles of FPCN subnetworks

**Figure 4:**
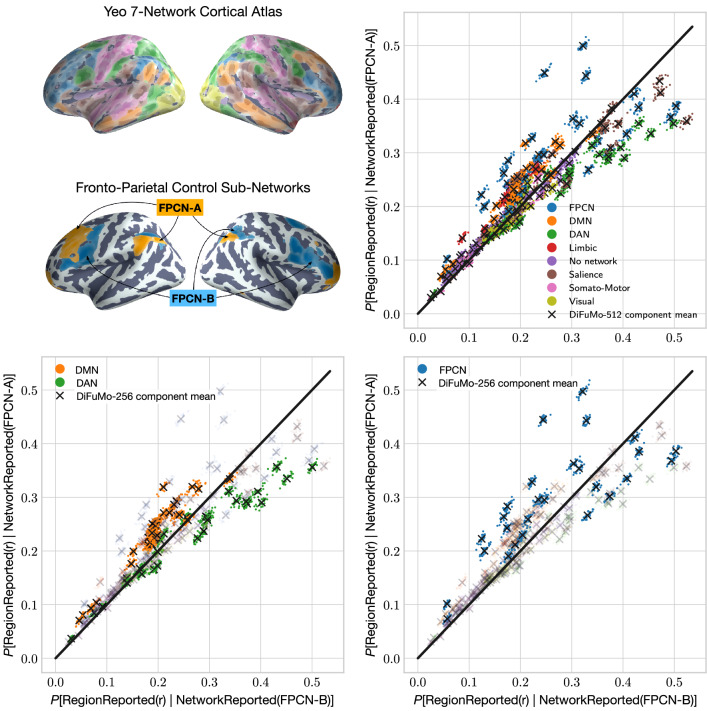
Comparison of the probabilities that DiFuMo-256 components coactivate with the two FPCN subnetworks. Regions are colored based on their network membership in the 17-Network brain atlas by Yeo et al.^22^. Only regions exhibiting a statistically significant ($p_\text {FDR} < 0.05$) coactivations with either subnetwork are included in the figure, based on the likelihood-ratio test and a correction for multiple comparison. $\mathbf{P} [ \text {RegionReported}(r) | \text {NetworkReported}(\texttt {FPCN-A}) ]$ denotes the conditional probability of region *r* being reported by studies reporting FPCN-A in the database. Probabilities are inferred in 1000 random 50% subsamples of the NeuroQuery CBMA database.

Recent findings suggest that the frontoparietal cognitive control network (FPCN) can be decomposed into sub-systems associated with disparate and overlapping mental processes. Dixon et al.^7^ studied two broad subsystems of the FPCN that also appear as separate networks in the influential 17-network model from Yeo et al.^22^. Using the same nomenclature, we label these two subsystems FPCN-A and FPCN-B. Dixon et al. observed preferential connectivity between FPCN-A and the default mode network (DMN), and between FPCN-B and the dorsal attention network (DAN). We reproduce these results by conducting a similar, but more compact, meta-analysis with NeuroLang.

For this analysis, we use the NeuroQuery^33^ database instead of Neurosynth. We express conditional probabilistic queries that include studies reporting activations in each of the two FPCN sub-networks. By contrasting their posterior probability maps, we identify a distinct meta-analytic connectivity pattern associated with each sub-network. Using the same probabilistic definition of network reported by studies as in the first example, we express a rule that calculates the coactivation pattern of each FPCN sub-network. In NeuroLang, we use the following rule to calculate the conditional probability of a region being reported given that a network is also reported
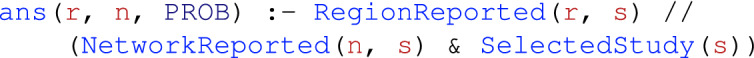
whose resulting ans table contains rows (*r*, *n*, *p*), where *p* is the probability of region *r* being reported active given that network *n*, where *n* is either FPCN-A or FPCN-B.

A likelihood-ratio test and an FDR correction ($\alpha = 0.05$) for multiple comparisons are used to identify statistically significant coactivating regions. To provide evidence that the results are not driven by one choice of studies, we estimate the conditional probabilities in 1000 random sub-samples of the NeuroQuery database (each sub-sample is 50% of the entire database). Note that statistical significance is determined in each of the 1000 sub-samples separately using the likelihood-ratio test.

In Fig. 4, we show scatter plots of the probabilities that each DiFuMo-256 brain region is active given activation of the FPCN-A or FPCN-B sub-networks are defined by Yeo 17-network parcellation. In the top right panel of Fig. 4, we show the results of regions that exhibit a statistically significant coactivation with at least one FPCN sub-network, based on a likelihood-ratio test. Statistical significance is assessed through sub-sampling of the NeuroQuery database In the left panel, regions are color-coded by their network membership according to the coarser Yeo 7-network parcellation to facilitate interpretation.

**Figure 5:**
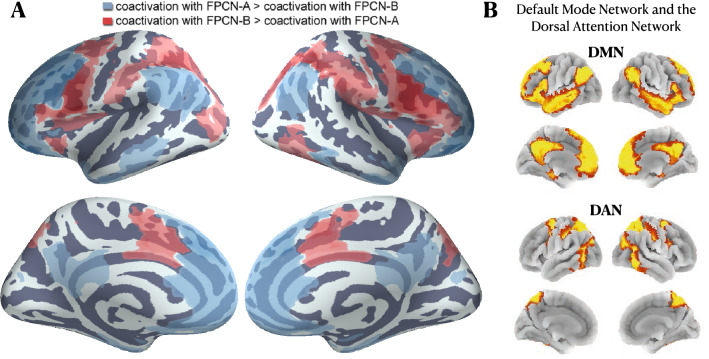
(**A**) DiFuMo-256 components that are more likely to coactivate with one FPCN sub-network than the other. In blue, we depict regions exhibiting a greater probability of coactivation with FPCN-A. In red, we depict regions exhibiting a greater probability of coactivation with FPCN-B. The absolute difference between region coactivation probabilities is defined as $\Delta = \mathbf{P} [ \text {RegionReported}(r) | \text {NetworkReported}(\texttt {{FPCN}-A}) ] - \mathbf{P} [ \text {RegionReported}(r) | \text {NetworkReported}(\texttt {{FPCN}-B}) ]$. A likelihood-ratio test and an FDR correction ($\alpha = 0.05$) for multiple comparisons are used to identify regions that exhibit significant coactivation with either network before estimating $\Delta$. (**B**) The default mode network (DMN) and the dorsal attention network (DAN) from the 7-Network atlas of Yeo et al. 2011. DMN regions are more likely to coactivate with FPCN-A, whereas DAN regions are more likely to coactivate with FPCN-B.

In general, regions belonging to the somatomotor, visual, and salience networks do not preferentially coactivate with either the FPCN-A or FPCN-B. In contrast, regions of the coarse FPCN show a dichotomy in their coactivations with either FPCN-A or FPCN-B. That is, meta-analysis supports the hypothesis that FPCN can be functionally divided into two sub-systems^7^. Importantly, we find a clearer dichotomy in the coactivation profiles of the DMN and the DAN with the FPCN sub-networks. On the one hand, 31 out of 32 DMN regions coactivate more with FPCN-A, while only one DMN region (a sub-region in the middle frontal gyrus) seem to exhibit a preferential coactivation with FPCN-B. In Fig. 5, we illustrate a meta-analytic coactivation contrast map between FPCN-A and FPCN-B, showing that the former coactivates to a greater extent with the core regions of the DMN, than does the latter. On the other hand, without indicating any preference, we observe that 21 out of 30 DAN regions exhibit statistically significant coactivations with FPCN-A, while 19 DAN regions show significant coactivations with FPCN-B. However, only 11 regions have a higher probability of activating, given an FPCN-B activation than FPCN-A, while the others have comparable probabilities of coactivating with either sub-networks. This is in line with the findings from Dixon et al.^7^, showing less distinction in the DAN with respect to coactivation with the FPCN sub-networks. Nonetheless, FPCN-B coactivates to a greater extent with the core regions of the DAN, the superior parietal lobule and frontal eye fields, than FPCN-A, as seen from the coactivation contrast map in Fig. 5.

## Discussion

We present a new domain-specific language (DSL), coined NeuroLang, which broadens the range of meta-analytic hypotheses that can be expressed and tested against an ever-increasing functional neuroimaging literature. Through probabilistic logic semantics, users can formally represent their hypotheses, query heterogeneous data, and reason about uncertainty in a unified language. Ultimately, NeuroLang is envisioned to lead a new generation of computational tools for neuroimaging data analysis, including meta-analysis, to reduce miscommunication in the community and promote formal and reproducible research. Although several probabilistic logic programming languages, such as ProbLog, already exist, we have chosen to design and implement Neurolang catering to application-driven features that are specific to the neuroimaging community. These include the use of aggregations and the possibility of in-language manipulation of probabilistic resolution results through probability encoding rules (PERs) that enable entire neuroimaging meta-analyses to be handled within the language. Other extensions which are not mentioned in this article such as handling hybrid open and close knowledge also motivate the development of NeuroLang. These can be seen in Zanitti et al.^26^. To support this novel language-oriented approach, we provide concrete neuroimaging meta-analysis examples fully performed with NeuroLang.

An important meta-analysis application, beyond finding consistent activation patterns, is inferring reliable and *specific* structure-function associations. Traditionally, researchers carefully select studies that “ask a similar question” or rely on databases of expertly curated annotations of studies, such as BrainMap^2^. However, non-automated meta-analysis is not scalable, time-consuming, and can suffer from low statistical power. As a response, automated tools have been developed, like Neurosynth^3^, NeuroQuery^33^, and more recently NiMare, to enable scalable, richer, and unbiased meta-analysis. However, as mentioned before, these tools cannot formally express nontrivial inclusion/exclusion criteria of studies to infer specific structure-function associations. In contrast, NeuroLang provides a unified formal framework to succinctly express versatile queries of functional specificity in the brain via *first-order logic semantics* rather than propositional logic statements^20^.

A recurring use-case of expressive querying throughout the examples is that of *segregation queries*, which we express using the first-order logical negation operator ($\lnot$) and existential quantifier ($\exists$). Using a segregation query enables a seamless split-up of studies in a meta-analysis while contrasting any number of topics and brain regions of interest both in forward and reverse inference paradigms. In this sense, segregation queries can enhance the specificity of inferred structure-function associations in brain regions that are putatively recruited to varying degrees by multiple tasks and brain networks, such as the VWFA^27^ or the anterior insula^34^ for instance.

The three networks example serves as a proof of concept for using segregation queries to derive specific structure-function associations for brain structures, such as networks. In this example, we infer the topics that are preferentially linked to each of the FPCN, DMN, and DAN. These three networks are known to exhibit competitive and cooperative coupling dynamics across a wide array of tasks^35^. Thus, observed activations may emerge from the dynamics of these networks depending on task demands^36^. This might yield in a blurry characterization of networks in terms of their specific roles and spatial arrangement, sometimes leading to nomenclature ambiguities across studies and research groups^37^. One way to solve this is to infer relatively specific functions of a network by “isolating” its activity pattern from the other networks. Achieving this isolation through segregation queries, we find preferential topic associations for the FPCN, DMN, and DAN that align with the general understanding of their roles^29,38,39^. Such a segregation-based meta-analysis can be performed in future studies, for instance, to create a foundation for a fine-scale taxonomy of brain networks depending on their inferred roles in addition to their connectivity patterns.

In the VWFA example, we use segregation queries to address controversial hypotheses^27,40^ about the role of this region in general visual processing beyond reading words. The main result is a statistically significant association for the VWFA with a topic loading on terms of object recognition given coactivation with attention but not language regions. However, the topic loads on terms such as ‘fusiform’ and ‘occipitotemporal’—zones in the close vicinity of the putative VWFA known to represent features and attributes of objects^41^, which might bias the results due to uncertainty in precisely locating the VWFA and the ambiguity in nomenclature across studies. But given that the dorsal attention network is active and its strong functional connectivity to the left occipitotemporal zone^40^, a link between the VWFA and “object recognition” is plausible. Concurrently, we observe no statistically significant topic associations for the VWFA when it coactivates with language but not attention regions. Yet, if we do not exclude studies reporting the coactivity among language and attention networks, a significant association with a topic related to “reading words” is observed. This finding suggests that the VWFA might not be specific to language per se, but rather have a broader role at the interface of language and visuospatial attention. Findings from^27^ suggest that the VWFA acts as a gateway between attention and language networks, such that the former amplifies the representations of written words so they may be conveyed to the latter. Our findings suggest that the VWFA is a more general visual processor that may be recruited in other visual tasks in addition to reading. This segregation-based meta-analysis can be extended to understand the dynamic roles of flexible regions in the brain, such as connector hubs.

In the third and fourth examples, we derive varying coactivation patterns within the FPCN revealing its heterogeneous organization. The FPCN comprises regions that coactivate across diverse conditions. Given the lack of formal definitions and fine lines between different executive functions, which are often conjointly studied, it can be difficult to determine domain-specific FPCN regions. As a potential solution to this problem, a segregation query can simultaneously select studies highly loading on topic terms related to a single function, discard studies loading on topic terms of other functions, and contrast them. The results reveal a relatively unique coactivation pattern *consistently* associated with each topic, consistent with findings of dynamical activity in canonical brain networks as a function of varying demands^42^. We say “relatively unique” because we only study a small set of topics for the sake of demonstration, while in fact there are putatively more functions attributed to the FPCN. This meta-analysis can include a cognitive ontology^43^ to systematically define *all* pertinent concepts and similarly contrast them^26^. Moreover, this type of meta-analysis can be effective in system-level causal modeling approaches (e.g., dynamic causal modeling)^44^, which require strong a priori hypotheses about the regions involved in particular contexts. Finally, we reproduce the results of Dixon et al.^7^, equivalently revealing two subsystems, FPCN-A and FPCN-B that exhibit distinct activations profiles and specific associations with the DMN and DAN, respectively. Although Dixon et al.^7^ have successfully performed this analysis using Neurosynth’s command line tools, we have been able to reproduce their findings with significantly more compact, declarative, and formal queries. In this sense, after representing the data in NeuroLang, a user only has to worry about what question to ask rather than about explicitly declaring every step needed to answer it.

Performing meta-analyses often requires integrating heterogeneous data. For example, Andrews et al.^11^ use a brain parcellation to characterize components of the DMN whose respective functions are decoded through reverse inference reasoning. Here, to study the functional profiles of brain networks and the coactivation patterns of FPCN subsystems, we integrate the DiFuMo-256 functional atlas. Components of this functional atlas overlap with anatomical landmarks whose names are used to label the components by experts to enhance interpretability^21^. The DiFuMo components are also grouped into 7 and 17 canonical networks whose labels have been integrated in our examples. This approach facilitated the formulation of hypotheses and the interpretation of results. We believe that future studies conducted with NeuroLang could benefit from its capacity to represent any anatomical and functional atlas as well as tabular meta-data and formal ontologies^26^. Moreover, it is imperative for a *complete* meta-analytic tool to be flexible enough to represent any type of parcellation, meta-analytic database, or, more generally, neuroscientific knowledge. Within our experiments, we have been able to represent both the Neurosynth database and its associated openly-shared topic models in NeuroLang. But, in other experiments, we use the NeuroQuery database because of its lower error rate in the extraction of peak activation coordinates^33^. Together, these examples demonstrate that NeuroLang is agnostic to the database used for conducting meta-analyses, and could incorporate future sources of neuroscientific knowledge with various topologies.

Of course, due to the analytical variability across neuroimaging studies^45^ and imperfections in data acquisition, knowledge representation should account for elements of uncertainty in the data. Probabilistic programs and databases constitute *general frameworks* for representing *structured* but *uncertain* knowledge. As these two paradigms reside at the heart of NeuroLang, uncertain data can be combined within its probabilistic programs. In our experiments, we model the reporting of functional modes and networks probabilistically based on their volumetric proportion that is reported by studies, although other indicators of uncertainty can be used. The probabilistic definition of uncertainty is *arbitrary*, but NeuroLang is expressive enough to represent any assumption. For instance, to obtain network-specific functional profiles, we combine meta-analytic data and functional atlases with data-driven topic models. The topics are associated with studies probabilistically by data-driven loadings from the fitted latent Dirichlet allocation model, which are based on the frequency of co-occurrence of terms in abstracts of studies^19^. Other measures of uncertainty can also be represented in NeuroLang, such as sample size, the relative location of peaks, study age, and methodological choices such as inference method, smoothing, and thresholding. Although mathematically modeling such information is not straightforward, the most important condition to incorporate them into uncertainty modeling is our ability to access them from the meta-data of each study. Latent information about studies cannot be easily modeled and hence accounted for by NeuroLang. For instance, a limitation of the current study is that some articles in the meta-analyses may report activations only within pre-defined ROIs, discarding those existing in other regions. Thus, it becomes impossible to differentiate articles that only report within ROI activations from those that genuinely did not observe activations in regions of no interest. This type of reporting bias can affect the resultant structure-function associations, artificially amplifying uncertainty and skewing the evidence in favor of specific structure-function associations. Although NeuroLang can essentially account for heterogeneous sources of uncertainty, it cannot readily take into account differences in methodological choices across studies, unless such information is accessible. Yet, even if such information is available, conceiving a mathematical model that robustly incorporates them into the analysis remains a challenging task. Another example of information not included in the meta-analytic databases is that within articles one-on-one associations between multiple regions/networks and topics may be reported. However, such finely-resolved information has not been automatically extracted by the scraping algorithms used by Neurosynth or NeuroQuery. As a result, these articles will be deemed non-specific, i.e. not relating a topic to a single network or vice-versa, and excluded in a segregation-based meta-analysis, as they violate the region-topic exclusivity condition within a single study. The problem lies not in NeuroLang, but in the assumption that a study is the smallest unit of analysis. Therefore, a segregation query will exclude any study that reports more than one network and more than one topic at a time. A plausible solution is to perform a nested meta-analysis, increasing the resolution to the level of contrasts or tables within a study. Yet again, such information must be available in databases to be adequately presented in NeuroLang.

It is worth noting that, so far, not all types of queries can be *efficiently* solved by NeuroLang’s engine. For example, *negated* disjunctive queries, such as $\lnot ( \text {FirstSegregation}(s) \vee \text {SecondSegregation}(s))$, where $\text {FirstSegregation}$ and $\text {SecondSegregation}$ correspond to two segregation rules—similar to those presented in our examples, can be computationally intractable in a voxel-level analysis, where $>150,000$ voxels need to be modeled. This is because extensions to logic programming, such as negation^46^, are not directly transposable to lifted query processing of unions of conjunctive queries on *probabilistic* databases^47^. In other words, more expressivity in a language leads to higher *data complexity*—the complexity of solving a query with respect to the size of the data^48^. In the case of whole-brain voxel-level statistical modeling, data complexity is significant, and solving a segregation query can become impractical due to the large number of voxels that are modeled. Efficient algorithms exist for solving a limited *subset* of queries on probabilistic databases^14^. NeuroLang privileges *lifted query processing* to solve queries on probabilistic databases (see the “Methods” section for details). Lifted query processing is an algorithm with a set of rules that translate a query to an algebraic expression to solve it in polynomial time. This allows NeuroLang to efficiently scale to large databases. However, to calculate the solution of a potentially non-liftable query, NeuroLang falls back to knowledge compilation strategies^49^. Yet, when modeling hundreds of thousands of brain voxels, we find resolution times of knowledge compilation to be impractical as well. Obtaining a solution for meta-analytic queries at the voxel level currently take several minutes to be solved with NeuroLang, while such queries can be solved in a few seconds by Neurosynth’s engine, which uses a custom python-based implementation. Nevertheless, major improvements to NeuroLang’s engine are currently underway.

In designing NeuroLang, we provide a high-level programming interface for harnessing meta-analysis databases in cognitive neuroscience research. We believe that this approach has *three* main advantages: Accessibility, Readability, and Sound semantics. Implementing a program to test a complex hypothesis against meta-analytic data can be time-consuming and error-prone, especially for those not proficient in general-purpose programming languages. A domain-specific syntax like NeuroLang’s eases the process of formulating hypotheses combining heterogeneous data. This has the potential of speeding up the meta-analysis process as well as making it highly reproducible. Moreover, being specific about the research question and assumptions in meta-analysis is an important practice^50^. NeuroLang’s logical syntax makes model assumptions and inclusion/exclusion criteria readable and understandable directly from the code of the program. Importantly, NeuroLang is grounded in formal mathematical logic, providing theoretical guarantees that both limit modeling errors and provide trust in the language. We believe that these advantages make NeuroLang a tool of choice for conducting functional neuroimaging analysis. Finally, studies performed using NeuroLang are highly reproducible. That is, a NeuroLang program used in a study in 2022 could be re-used in another study in 2027, 5 years later, either as a modeling inspiration for an entirely different study or to confront the original study’s findings with results published from 2022 to 2027.

## Methods

We apply a language-oriented programming approach to the problem of expressing and testing meta-analytic neuroscientific hypotheses. That is, instead of using a general-purpose programming language to solve the problem, we design a domainspecific language (DSL) to represent the problem, and solve it in that language. NeuroLang uses *logic*, *declarative*, and *probabilistic* programming paradigms. We start by introducing computer science concepts required to understand the semantics of the language. Then, we explore how these formalisms can be applied to the specific case of expressing cognitive neuroscience hypotheses. Finally, we detail the technicalities of solving queries on NeuroLang programs when working at the whole-brain neuroimaging scale.

### Probabilistic logic programs and databases

**Figure 6:**
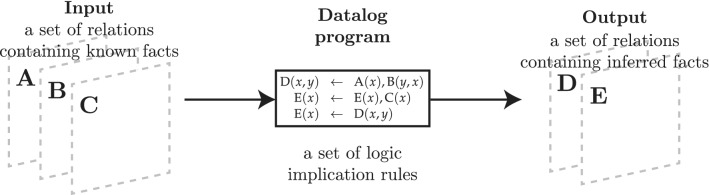
Deductive inference in Datalog. Using knowledge and implication rules to infer new knowledge.

To *represent* heterogeneous neuroscientific knowledge, NeuroLang leans on Datalog^25^, a fully declarative logic a programming language designed to efficiently solve queries on large deductive databases. A Datalog rule takes the form $\forall \mathbf {x}, \left( \psi (\mathbf {x}) \leftarrow \exists \mathbf {y}, \varphi (\mathbf {x}, \mathbf {y}) \right)$, where $\mathbf {x}$ is a set of universally quantified variables, $\mathbf {y}$ is a (possibly empty) set of existentially quantified variables, $\psi$ is a relational symbol, and $\varphi (\mathbf {x}, \mathbf {y})$ is a conjunctive logic formula over $\mathbf {x} \cup \mathbf {y}$. For readability, the implicit $\forall$ and $\exists$ quantifiers are often omitted, and the rule is written $\psi (\mathbf {x}) \leftarrow \varphi (\mathbf {x}, \mathbf {y})$. A Datalog program is a set of such rules. The input of the program is a set of *extensional* facts and its output is a set of *intensional* facts that have been obtained through deductive inference, based on the program’s rules and input facts. This process is summarised in Fig. 6. In NeuroLang, we write a Datalog rule $\forall (x, z), \left( P(x, z) \leftarrow \exists y, Q(x, y, z) \wedge R(z) \right)$ as . Theoretical formalisms and efficient query resolution algorithms have been developed by the logic programming community over the past decades. By restricting the syntax of its rules, Datalog queries have been proven to be solvable in polynomial time w.r.t. the size of the database^25^. Strong guarantees on query resolution complexity are primordial to handling high dimensional whole-brain neuroimaging data.

**Figure 7:**
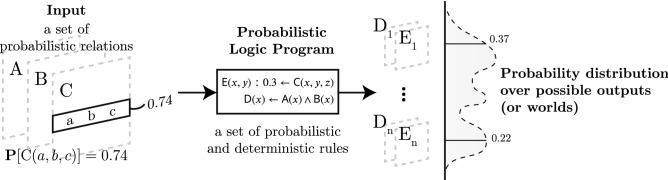
A probabilistic logic program defines a probability distribution over its possible outcomes. Note that the probabilities and the distribution are arbitrary and are used for illustration only.

Testing cognitive neuroscience hypotheses require aggregating data from many subjects or studies using statistical models. Logic programming languages were not designed for statistics or probabilistic modeling, as their programs live in a world where everything is either deterministically true or false and only one outcome is possible. To address this limitation, logic programming languages were extended with probabilistic semantics to incorporate uncertainty and allow for probabilistic inference, such as ProbLog2^51^ or CP-Logic^52^. Following ProbLog’s semantics, albeit with slightly different syntax, a probabilistic rule $\psi (\mathbf{x} ) : \alpha \leftarrow \varphi (\mathbf{x} )$ describes that the fact $\psi (\mathbf{x} )$ is true with probability $\alpha$, whenever the deterministic predicate $\varphi (\mathbf{x} )$ is true. Thus, probabilistic rules describe non-ground probabilistic facts. Probabilistic logic programs define a distribution over possible outputs of the programs’ execution, i.e. *possible worlds*^53^. A schematic summary of probabilistic logic programming is illustrated in Fig. 7. For a more detailed introduction to probabilistic logic programming, we refer the reader to De Raedt et al.’s review^54^. In NeuroLang, we write a probabilistic rule $P(x, y) : f(x, y) \leftarrow Q(x, y) \wedge R(y)$ as
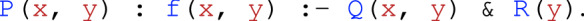

Inferring the probability of a query boils down to summing the probabilities of all possible worlds where this query is verified (see Fig. 7). This process is called *weighted model counting*^55^. The number of possible worlds is often very large, and naively counting possible worlds become intractable. For example, if we model the activation of each brain voxel as independent Bernoulli random variables, the number of possible worlds would be $2^K$, where *K* is the number of voxels in the brain, typically numbered in the hundreds of thousands. Solving weighed model counting problems on real-world data requires efficient resolution algorithms. Knowledge compilation finds compact representations of large probabilistic programs and can be used to solve queries drastically faster^14,56^.

**Figure 8:**
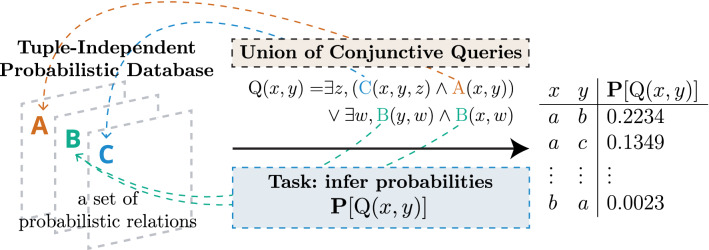
Solving a union of conjunctive queries on a tuple-independant database.

In parallel, the field of probabilistic databases extended traditional relational databases with the possibility of encoding uncertain knowledge using probabilities^57^. A probability can be attached to any tuple in the database, as illustrated in Fig. 8. Similarly to probabilistic programs that define a distribution over possible outputs, a probabilistic database defines a probability distribution over a set of possible databases (or worlds), where tuples are chosen to be true or false based on their probability. The probability attached to a tuple then corresponds to the marginal probability of that tuple being found in any database randomly chosen from the distribution over possible databases. Probabilistic tuples within the database are often assumed to be independent random events, in which case the database is called a tuple-independant database (TID). A tuple with probability 1 is true in all possible databases, and a tuple with probability 0.5 is true in half of the possible databases.

**Figure 9:**
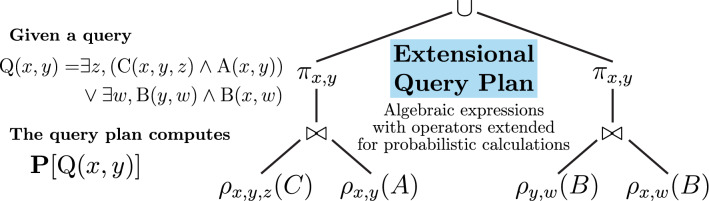
An extensional query plan solves $\mathbf{P} [Q]$ for a given query *Q* by using algebraic operations that are extended with probabilistic calculations^14^. Symbols $\rho$, $\pi$ and $\bowtie$ refer to the operations of *rename*, *projection* and *natural join* as they are used in database theory, specifically in relational algebra^25^.

In some cases, solving a query on a probabilistic database can be done much more efficiently than through knowledge compilation approaches. One recent theoretical result is the *dichotomy theorem*, which classifies queries on TIDs based on their complexity^13^. At the heart of this theorem, there is a resolution strategy named *lifted query processing*. It applies, based solely on syntactic analysis of queries, a set of rules that derive an algebraic expression that computes the probability of the query^14^. We illustrate this process in Fig. 9. Liftable queries have a polynomial data complexity. If the rules fail to apply, the query is said to be *non-liftable*, and has been proven to have a #P-hard complexity. In NeuroLang, the query preprocessing engine is in charge of choosing the best algorithm to solve each query: for probabilistic queries, if the query is liftable according to Dalvi & Suciu’s dichotomy theorem^13^, then the lifted query algorithm is applied; otherwise, the query is compiled to an SDD representation and model counting is applied^14^. According to the dichotomy theorem, a query is said to be liftable if it belongs to the class of union of conjunctive queries using a single quantifier, atomic negation, and each predicate appears either in positive or negative form; furthermore, the query needs to admit a translation into a provenance relational algebra query where the probabilities can be then purely calculated through relational algebra operations, i.e. a safe plan. We refer the reader to articles such as Van der Broeck and Suciu^14^ for a more technical description. As an example of a non-liftable query according to Dalvi and Suciu^13^, the last query of the “Meta-analysing the role of the visual word-form area in attention circuitry” cannot be lifted because it is not a disjunctive query, it has 2 quantifiers: an existential in *s* and a *universal one* in *n2* as a consequence of the negated existential.
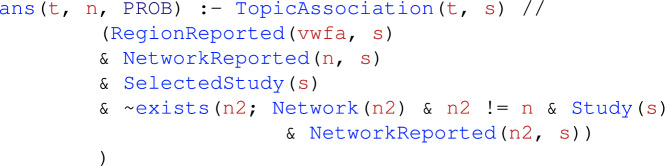


By incorporating probabilistic semantics and fast query resolution algorithms from both probabilistic logic programming and probabilistic databases, NeuroLang is a full-fledged *probabilistic* programming language^20^. This approach makes it possible to express a wide variety of programs and queries, some of which can be efficiently solved using lifted query processing on probabilistic databases, even at the voxel level.

### Syntactic specificities of NeuroLang

We describe the syntactic extensions of typical logic and probabilistic logic programming languages that we made to provide features necessary to express end-to-end meta-analyses in NeuroLang.

**Figure 10:**
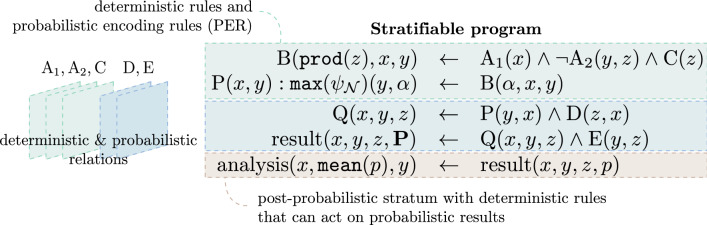
Stratification in NeuroLang. The program’s input contains both deterministic ($\text {A}_1$, $\text {A}_2$, and $\text {C}$), and probabilistic ($\text {D}$ and $\text {E}$) tables.

A NeuroLang probability encoding rule (PER) captures the result of a probabilistic inference into a deterministic table. They are a syntactic convenience, or *sugar syntax*, that makes it possible to solve probabilistic queries within the program and process their solution with deterministic rules. Internally, we *stratify* (i.e. split in several code sections) a NeuroLang program as deterministic and probabilistic *strata*. Stratification allows us to *programmatically* post-process and analyzes results from probabilistic calculations within the same self-contained program, as illustrated in Fig. 10. Moreover, this strategy can be used to supplement deterministic strata with logic programming extensions that would not necessarily be compatible with probabilistic programming^26^.

PER can either infer *marginal* or *conditional* probabilities. A marginal PER takes the form
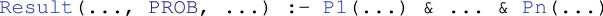
where P1, ..., Pn are deterministic or probabilistic relational symbols, and where PROB is a special symbol that indicates the position of the attribute where the probability resulting from the probabilistic query represented by the rule’s body, marginal or conditional, will be reified. We refer the interested reader to Zanitti et al.^26^ for a detailed description of the semantics of PERs. All other variables present in the head of the rule have been replaced with three points “...” to simplify the example. A conditional PER is only slightly different in that it calculates the probability of the conjunction of literals being true, *given that* another the conjunction of literals is true. Conditional PER take the form
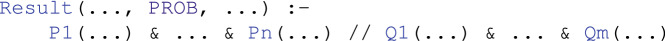
where the // operator applies a *probabilistic conditioning*. The PROB attribute of the resulting Result relation encodes the conditional probabilities $\mathbf{P} [ \text {P}_1(\mathbf {x}) \wedge \cdots \wedge \text {P}_n(\mathbf {x}) | \text {Q}_1(\mathbf {x}) \wedge \cdots \wedge \text {Q}_m(\mathbf {x}) ]$, for each tuple $\mathbf {x}$ such that the probability is strictly positive. This is a brief description of PER and it is beyond the scope of this paper to formalize its definition. For a more detailed description, please refer to Zanitti et al.^26^.

NeuroLang supports existential quantification of variables using rules such as , where  is existentially-quantified using the language’s special symbol . The language also supports negation within its deterministic and probabilistic rules and aggregations within its deterministic rules. Aggregations are functions that operate on multiple tuples or rows that are grouped together. Specifically, Neurolang admits aggregations only on deterministic nonrecursive rules. An example of an aggregation rule is , which counts, for each possible assignment of *x*, the number of grouped tuples (*y*, *z*) such that *Q*(*x*, *y*, *z*) is true, and stores this count in the second column of table *P*. Probabilistic tables can be constructed dynamically from deterministic rules. For example, the rule  constructs a probabilistic table *P*, assigning a probability to each tuple (*x*, ) based on function *f*. In the case where such rule generates multiple probabilities for the same tuple (*x*, ), an error is thrown and the user is advised to either change their probabilistic definition or apply an aggregation function on the probabilities, such as . The head of these rules must be deterministic.

### Likelihood ratio test for NeuroLang queries

Throughout this work, we express meta-analytic conditional probabilistic queries of the form $\mathbf{P} [\varphi (s)|\psi (s)]$, where $\varphi (s)$ and $\psi (s)$ are first-order logic formulas describing study-specific probabilistic events of interest; such as whether a region/network is reported by study *s*, or whether *s* is associated with a topic related to a particular psychological concept. For brevity, we write $\mathbf{P} [\varphi (s)|\psi (s)]$ instead of $\mathbf{P} [\varphi (s) = \top | \psi (s) = \top ]$, where $\varphi (s)$ and $\psi (s)$ are modeled as Bernoulli random variables that have a probability of being true ($\top$) or false ($\bot$) in any possible execution of the probabilistic logic program. The formula $\psi (s)$ imposes conditions that select studies that will be included in a meta-analysis. To test the statistical dependence of $\varphi (s)$ on $\psi (s)$, we use a likelihood ratio test, whose null ($H_0$) and alternative ($H_1$) hypotheses are

1 $$\begin{aligned} H_0 :&\quad \mathbf{P} [\varphi (s)|\psi (s)] = \mathbf{P} [\varphi (s)|\lnot \psi (s)] = \mathbf{P} [\varphi (s)] \end{aligned}$$

2 $$\begin{aligned} H_1 :&\quad \mathbf{P} [\varphi (s)|\psi (s)] \ne \mathbf{P} [\varphi (s)|\lnot \psi (s)] \end{aligned}$$

We define the likelihood ratio as $\lambda \triangleq \mathcal {L}(H_1) / \mathcal {L}(H_0)$, where $\mathcal {L}(H_1)$ and $\mathcal {L}(H_0)$ are the maximum likelihood of the observed data under the alternative and null hypotheses defined as

3 $$\begin{aligned} \mathcal {L}(H_0)&\triangleq \text {Bin}(k; n, \mathbf{P} [\varphi (s)]) \text {Bin}(m - k; N - n, \mathbf{P} [\varphi (s)]) \end{aligned}$$

4 $$\begin{aligned} \mathcal {L}(H_1)&\triangleq \text {Bin}(k; n, \mathbf{P} [\varphi (s)|\psi (s)]) \text {Bin}(m - k; N - n, \mathbf{P} [\varphi (s)|\lnot \psi (s)]) \end{aligned}$$

where *m* is the number of studies *s* such that $\varphi (s)$, *n* is the number of studies *s* such that $\psi (s)$, *k* is the number of studies *s* such that $\varphi (s) \wedge \psi (s)$, and *N* is the total number of studies within the database. As $2\log \lambda$ is asymptotic $\chi ^2$ distributed with 1 degree of freedom^58^, it provides an estimate of the false-positive rate when rejecting the null hypothesis.

## Supplementary Information

Supplementary Information.

## Data Availability

All data and code are available to be openly used by the community. Neurosynth and NeuroQuery data are respectively found at https://github.com/neurosynth/neurosynth-data and https://github.com/neuroquery/neuroquery_data. The analysis scripts will be stored in a shared public repository along with the source code of NeuroLang on GitHub at https://github.com/NeuroLang/NeuroLang.
